# Role of Medicinal Plants and Natural Products on Osteoporotic Fracture Healing

**DOI:** 10.1155/2012/714512

**Published:** 2012-09-02

**Authors:** Mohd Azri Abd Jalil, Ahmad Nazrun Shuid, Norliza Muhammad

**Affiliations:** ^1^Department of Pharmacology, Faculty of Medicine, Universiti Kebangsaan Malaysia, Jalan Raja Muda Abd Aziz, 50300 Kuala Lumpur, Malaysia; ^2^Department of Basic Medical Science, Kulliyyah of Nursing, International Islamic University Malaysia, Level 2, Jalan Hospital Campus, P.O. Box 141, Pahang Darul Makmur, 25710 Kuantan, Malaysia

## Abstract

Popularly known as “the silent disease” since early symptoms are usually absent, osteoporosis causes progressive bone loss, which renders the bones susceptible to fractures. Bone fracture healing is a complex process consisting of four overlapping phases—hematoma formation, inflammation, repair, and remodeling. The traditional use of natural products in bone fractures means that phytochemicals can be developed as potential therapy for reducing fracture healing period. Located closely near the equator, Malaysia has one of the world's largest rainforests, which are homes to exotic herbs and medicinal plants. *Eurycoma longifolia* (Tongkat Ali), *Labisia pumila* (Kacip Fatimah), and *Piper sarmentosum* (Kaduk) are some examples of the popular ethnic herbs, which have been used in the Malay traditional medicine. This paper focuses on the use of natural products for treating fracture as a result of osteoporosis and expediting its healing.

## 1. Fracture

Fracture is defined as a complete or incomplete separation in the continuity of the bone [1]. Fracture healing is a complex physiological process that involves the coordinated participation of hematopoietic and immune cells within the bone marrow. In conjunction with vascular and skeletal cell precursors, it also includes mesenchymal stem cells (MSCs), which are recruited from the circulation and the surrounding tissues [2, 3].

The two basic types of fracture healing are the primary or direct fracture healing and the secondary or indirect fracture healing. Primary (direct) fracture healing occurs with very minimal callus formation. It is a direct attempt of bone to reestablish its continuity and thus requires direct contact of cells in the cortex [4]. Primary healing occurs rarely as the majority of fracture repairs undergo secondary or indirect healing [5]. Based on the histological observations, secondary fracture healing occurs in four overlapping phases, which are haematoma formation, early inflammatory (two to four weeks), repair (proliferation and differentiation, within a month or two), and late remodelling phase (lasting for months or years) [6]. 

The clinical impact of fractures is substantial. Significant pain, disability, and deformity will trail following a fragility fracture. If the fracture union is not achieved, the patient may suffer long-term disability. Degenerative joint disease distal to the fracture and reflex sympathetic dystrophy are other recognized complications [7]. Hip fractures, even less common than vertebral fractures, contribute to the majority of the mortality, morbidity, and costs associated with osteoporosis [8]. Osteoporotic fractures are still an unsolved problem to the surgeon as well as for the patient. There are two ways of improving the fracture healing process: first, the developments of special implants to avoid implant failure; second, the improvement of bone quality to speed up and improve callus formation and otherwise to biologically advance implant fixation [9]. 

## 2. Osteoporosis

Osteoporosis is a heterogeneous cluster of abnormal processes characterized by the net loss of bone. It results in a decrease in total mineralized bone without a decrease in the ratio of bone mineral to the organic matrix [10, 11]. As a result, there is a decrease in the overall amount of bone. The bone loss affects both cortical and trabecular bone, with trabecular bone loss more predominant in postmenopausal osteoporosis. Consequently, osteoporosis would lead to a bone with less tensile strength and significantly more susceptibility to fracture with less force [10]. This syndrome is clinically silent but progressive, usually only noted when a fracture occurs [12]. It is one of the most major public health problems with a mortality of 30% in the first year following the osteoporotic hip fracture [13]. 

During the early menopausal years in women, there is a dramatic reduction in circulating estrogen. As a result, there is an increase in the rate of bone resorption, but not reformation. This creates an imbalance and sets the stage for osteoporosis [14, 15]. Although bone loss in women slows after the early postmenopausal years, loss continues through the latter decades of life, and in very old age the rate of loss increases again [16, 17]. In addition to hormonal changes, age-related bone loss is also due to reduced ability to utilize calcium [18], decreased vitamin D supply due to lower production and reduced absorption, and decreased activation of vitamin D by the kidneys [19, 20]. All of these factors contribute to the increase with age in another hormone—parathyroid hormone [21, 22]. When there is too much parathyroid hormone released in the body (hyperparathyroidism), bones release excessive calcium into the blood stream. As a direct result, bones lose their density and hardness.

## 3. Osteoporosis in Male

Although traditionally regarded as a disease of women, especially after menopause, osteoporosis also occurs frequently in men. Men steadily lose bone mineral density with aging, and one in five men over 50 will suffer an osteoporotic fracture [35]. Almost 30% of all hip fractures are in men, and the mortality following a hip fracture is substantially higher in men than in women [36]. Men represent between 20% and 40% of all patients with each types of fracture that frequently affect the hip, vertebrae, forearm (wrist), and humerus. These fragility fractures may occur following minimal trauma such as a fall from standing height [37]. 

Bone loss in men has many causes, and often the same patient is affected by several of these. As in women, primary osteoporosis includes age-dependent and idiopathic osteoporosis. Age-related bone loss is dependent on estrogen production in both sexes, not only in women [38, 39]. While estrogen is considered the female sex hormone, men also produce some estrogen. And as they age, men may experience a decrease in their ability to convert male sex hormones called androgens into estrogen [40, 41]. Recent research suggests that estrogen deficiency and reduced levels of other sex hormones may be a cause of osteoporosis in men. The main causes of secondary osteoporosis in men are excessive alcohol use, treatment with glucocorticoids, and hypogonadism, including that experienced by men receiving androgen deprivation therapy (ADT) for prostate cancer [42].

It is impossible to reverse the osteoporosis to its original form; it is just possible to prevent deterioration. Analgesic drug, heat, massage, and rest can be used to relief the osteoporotic pain [43]. The use of calcium, estrogen, calcitonin and vitamin D has been recommended too. Recently, few studies concerning the role of antioxidants in osteoporosis have been published, and the results show that there is a correlation between antioxidants and osteoporosis. Free radicals play an important role in many diseases such as diabetes, degenerative disorders, and cancer [44]. In normal conditions, there is a balance between free radicals and antioxidants defensive system. Sometimes, this balance is lost, which is called oxidative stress. Oxidative stress has been the centre of attention in recent studies of osteoporosis pathogenesis [45]. 

## 4. Importance of Natural Product

Today, it is estimated that about 80% of individuals in the developing countries still rely on traditional medicine-based largely on plants and animals for their primary health care. Herbal medicines are currently in demand, and their popularity is increasing day by day [46]. Herbal drugs are fairly preferred due to their effectiveness, fewer side effects, and relatively low cost [47]. It also has a brighter prospect in the global market. The market for ayurvedic medicines is estimated to be expanding at 20% annually [48]. 

Due to some adverse effects or lack of efficacy of synthetic drugs, the potential efficacy of traditional medicines has stimulated the interest of scientists and doctors to turn on to traditional medicines for treatment of some chronic and difficult diseases, including the treatment for osteoporosis [49]. In traditional Chinese medicine (TCM), osteoporosis is classified as “rheumatism involving the bone” and “atrophic debility of bones” [50]. Based on the theory of “Kidney dominates bone” in TCM, many medicinal herbs have been prescribed to treat bone metabolic disorders for long time [51]. 

The *Huang Di Nei Jing *(*The Yellow Emperor's Classic of internal Medicine*) says, “The Meridians move the qi and blood. As a result, tendons and bones get nourished whereas joints get facilitated.” *Astragalus membranaceus *Bge is one of the most popular herbs for restoring Qi and also can be applied for the osteoporosis treatment. The water extract of the root of *A. membranaceus *prevented osteoporosis induced by dexamethasone or by ovariectomy in rats by inhibiting osteoclast, decreasing bone absorption and promoting bone formation [23]. In TCM, *Curculigo orchioides *Gaertn., *Epimedium grandiflorum *Morr., *Morinda officinalis *How., *Cistanche salsa *G., *Eucommia ulmoides *Oliv., *Psoralea corylifolia *L., *Cuscuta chinensis *Lam., and *Dipsacus japonicus *Mip. possess the efficacy of tonifying kidney, strengthening Yang, and strengthening tendons and bones [24]. On the other hand, Fructus Ligustri Lucidi (FLL, Chinese name, Nvzhenzi) is one of the examples of herbs for nourishing *Yin *due to estrogen deficiency [52]. FLL can act on pituitary gland and further modulate endocrine function [53] and also useful in the prevention of bone marrow loss in cancer patients receiving chemotherapy [54]. 

The term “ayurveda” can be defined as knowledge *(veda) *of the lifespan *(ayu). *This knowledge is recorded in the ancient literature of India, referred to collectively as the Veda [55]. Ayurvedic medicine is based on breathing techniques, meditation, and yoga [56]. In Ayurvedic culture, the young bark of *Ficus religiosa *(family-Moraceae) also known as Ashwatha or Ashvattha, has been widely used in the treatment of bone fracture [27]. The stem bark cleaned with urine of a boy (below 7 years old) is taken and ground. Two spoonfuls of paste were administered twice daily for 21 days. Paste is also applied on the affected part and bandaged [29]. *Cissus quadrangularis *(family-Vitaceae), commonly known as the Asthishunkala, is an indigenous medicinal plant of India [57]. Methanolic extract of Cissus quadrangularis has been reported to promote the healing process of experimentally fractured radius-ulna of dogs, proven by radiological and histopathological examinations [28]. 

The native of Eastern Ghats (India's eastern coast area) use different types of plants to treat bone fractures. According to Eastern Ghats folklore, *Alangium salvifolium*, *Christiella subpubescens*, *Diospyros chloroxylon*, *Erythrina fusca*, *Mimosa intsia*, *Phoenix loureiroi*, and *Rhaphidophora pertusa* may be used for the treating bone fracture [29]. The bark of Symplocos loba is used externally for poulticing fractures, as it promotes the healing of bones [30]. *Oryza sativa* Linn (family-Poaceae), commonly known as Asian rice, is used in fracture healing and as a poultice to reduce inflammation at the affected areas [31].  Table 1 reveals the earlier studies on traditional medicinal plants by different cultures and their usage part.

## 5. Malay Traditional Medicine

The principles of Malay traditional medicine are generally based on the Arabic *Unani *medicine and Galenic philosophy in addition with other practices of Indonesian, Chinese, Indian, and *orang asli *(indigeneous people) traditional medicines [58]. It consists of chants (*jampi*), prayers (*doa*), massage, abstinence (*pantang*), and other practices, plus various natural resources from plants, animals, microorganisms and minerals for the purpose of treating and preventing illnesses, and for rehabilitation and health promotion. Medications containing single or compound medicinal plants may be dispensed in many forms, such as powders, capsules, pills, *makjun*, medicated oils, simple distillates, decoctions, infusions, paste, and poultices. Documentation of Malay TM practices is rather scarce. Most practices rely on old references, such as *Mujarabat Melayu*, *Tajul Muluk*, *Tajus as Salatin, *and *Surat Tib Ubat* [33]. The earliest scripts on the ethno-botanical uses of Malaysian plants dated back to the time of British colonialism. Some of these were publications [59, 60] providing monumental references for researchers on the utilization of medicinal plants in Malay traditional medicine.

## 6. Tongkat Ali

*Eurycoma longifolia *(EL) is a traditional medical plant known as *Tongkat Ali* in Malaysia, *Tung Saw* in Thailand, and *Pasak Bumi *in Indonesia. The plant is under the family of *Simaroubaceaea*. The water decoction of its root is a well-known folklore medicine to enhance sexuality, fertility, and antiaging [61]. The herb contains Quassinoid alkaloid with properties curing Malaria, allergies, alleviating fevers and reduced tumors. The water-soluble extract contains among other things, tannins, high molecular weight polysaccharides, glycoproteins, and mucopolysaccharides [62]. 

Studies have revealed that *EL *contain *eurycomanone, eurycomanol, eurycomalactone, *and alkaloid that may help to increase the free testosterone level in the blood and also inhibit sex hormone binding globulin [63]. Testosterone has been approved to preserve bone mass development. Therefore, testosterone can help to prevent osteoporosis. Testosterone replacement could increase the mass and density of the bone and become efficient treatment for osteoporosis [64]. The main treatment to prevent diseases related to testosterone deficiency is hormone replacement therapy whereby testosterone is injected intramuscularly [65]. Some patients may refuse this treatment because of the painful administration of testosterone and its associated adverse effect, especially prostate cancer. Tongkat Ali can be used as an alternative to increase the testosterone level since *EL *is believed to have proandrogenic effect [66]. 

 A study has shown that EL has a great potential as an alternative agent to testosterone replacement in treating androgen-deficient osteoporosis in men. It has good safety profile and convenient oral route of administration [32]. The active compound in *EL *can increase the level of testosterone in blood. The increase of testosterone can induce the androgen receptor, which is located in osteoblast and osteoclast cell. Testosterone and 5-*α*-dihydrotestosterone can inhibit *receptor activator of nuclear factor kappa-*β* ligand (*RANKL) and colony-forming unit-macrophages and further reducing osteoclast numbers [67]. As a result, the bone resorption process will be decreased and thus bone mass density will be maintained. These properties are essential in bone fracture healing process.

## 7. Kacip Fatimah

Kacip Fatimah, also known as *Labisia pumila *(LP), is a member of small genus of slightly woody plants of the family Myrsinaceae [68, 69]. The locals know it by the name as *Selusuh Fatimah, Rumput Siti Fatimah, Akar Fatimah, Pokok Pinggang, *and* Belangkas Hutan* [60, 70]. Its water extract is traditionally consumed especially by the Malay women to treat menstrual irregularities and painful menstruation, help contracting birth passage after delivery, and to promote sexual health function [71]. It has also been used to treat dysentery, gonorrhoea, rheumatism, and sickness in bones [72, 73]. 

Postmenopausal women are prone to osteoporosis due to the reduction in estrogen level. Estrogen can induce osteoclasts apoptosis and inhibit osteoblasts apoptosis, which will indirectly reduce bone resorption and increase bone-formation activity [74]. Proinflammatory cytokines such as, IL-6 and IL-1 may influence osteoclastogenesis by stimulating self-renewal and inhibiting the apoptosis of osteoclasts progenitors [75, 76]. They promote osteoclasts differentiation which is an important stimulator of bone resorption that has been linked to accelerated bone loss seen in postmenopausal women [74]. Estrogen is able to suppress the production of these proinflammatory cytokines. LP, which has been opposed to exert phytoestrogen property, can be used as an alternative to estrogen replacement therapy (ERT) in postmenopausal inflammation-induced osteoporosis. In contrast to ERT, which can cause many harmful side effects, LP, which originated from natural resources, will not cause any side effect, if taken within its safe therapeutic dose [77]. 

Based on previous studies, LP has been shown to exhibit antioxidative properties due to the presence of flavanoids, ascorbic acid, beta-carotene, anthocyanin, and phenolic compounds [78, 79]. Flavonoid has been shown to be highly effective scavenger of free radicals that are involved in diseases such as osteoporosis and rheumatism, which is associated with aging due to oxidative stress [80]. Anthocyanin and phenolic, on the other hand, not only play a role as antioxidant agents, but also as anti-inflammatory agents [52, 81, 82]. These antioxidative and anti-inflammatory properties of LP extract explained the effectiveness of this medicinal plant against various diseases such as osteoporosis, rheumatism, and women sexual function.

## 8. Kaduk

*Piper sarmentosum *(PS) (*Piperaceae, *Malay name: *Daun Kaduk*) is a creeping shrub with erect branchlets that can grow up to 20 cm. In Malay culture, the water decoction of its leaves is being used for treating diabetes, hypertension, cough, and joint aches [83]. The extract of the different parts of PS plant is known to possess benefits. It also possesses antioxidant, antiplasmodial, antituberculosis, anti-inflammatory, anticarcinogenic, and hypoglycemic properties [84, 85]. The methanolic extract of PS consists of a high natural antioxidant scavenger, naringenin, a flavonoid group with high superoxide scavenging activity [83]. Past experimental studies showed that flavonoids prevented ovariectomy-induced osteopenia and strengthened bone in ovariectomised animals [86]. Therefore, fracture healing properties of PS may be attributed to the action of flavonoids present in the PS extract.

A previous study on experimental fractured animal models revealed better fracture healing following PS administration during the late phase of fracture healing [34, 87]. In addition, Ima-Nirwana et al. [26] had observed an antiosteoporotic effect of PS aqueous extract in the adrenalectomised rats. The beneficial effect of PS on osteoporosis and fracture healing is most probably attributed to the antioxidative actions of the PS flavonoids, which may prevent oxidative stress [26]. Previous experimental studies on animals confirmed that osteopenia following oestrogen loss can be prevented by the supplementation of antioxidants [88]. It has also been found that glutathione peroxidase, an antioxidant enzyme secreted by osteoclasts, has a major role in reducing H_2_O_2_ [89]. Hence, supplementation of antioxidants can strengthen the bone and promote fracture healing in osteoporotic patients [90]. PS is rich in a natural antioxidant superoxide scavenger (Naringenin), which may have beneficial effect in promoting fracture healing most probably by reducing ROS through its free radical-scavenging activity. Hence, PS may have the potential to be added as antioxidant supplements to the current treatment modalities.

## 9. Conclusion

As a conclusion, a serious concern should be taken on osteoporosis. Because of the dormant properties of the disease, it is hard to recognize the symptoms until fracture occurs. Several studies have shown that androgen deficiency can lead to osteoporotic fractures. Therefore, various treatments should be considered to promote the healing period of the fracture. Natural products could be considered as a natural heritage from Mother Nature as a source of medicine. Thus, more extensive studies should be conducted to explore the healing properties of different types of medicinal plants to produce an alternative and effective treatment for the osteoporotic patient. A few of Malay's famous medicinal plants like EL, LP, and PS, can be used to enhance fracture healing. The plants possess the androgen-like and antioxidative properties, which are important in the promotion of bone fracture healing.

## Figures and Tables

**Table 1:** Table revealing earlier studies on traditional medicinal plants and their usage part.

Plants	Part used	System of medicine	References
*Astragalus membranaceus* Bge	Roots	Chinese	[23]
*Curculigo orchioides *Gaertn	Rhizome	Chinese	[24]
*Epimedium grandiflorum *Morr.	Roots and leaves	Chinese	[24]
*Morinda officinalis *How.	Root	Chinese	[25]
*Cistanche salsa * G. Beck	Stem	Chinese	[25]
*Eucommia ulmoides *Oliv.	Bark	Chinese	[26]
*Psoralea corylifolia *L.	Fruit	Chinese	[25]
*Cuscuta chinensis *Lam.	Seed	Chinese	[25]
*Dipsacus japonicus *Mip.	Root	Chinese	[24]
*Fructus Ligustri Lucidi*	Fruit	Chinese	[24]
*Ficus religiosa*	Young bark and stem bark	Ayurveda	[27]
*Cissus quadrangularis*	Stems	Ayurveda	[28]
*Alangium salvifolium*	Root bark	Ayurveda	[29]
*Christiella subpubescens*	Rhizome and tuberous	Ayurveda	[29]
*Diospyros chloroxylon*	Stem bark	Ayurveda	[29]
*Erythrina fusca*	Seed	Ayurveda	[29]
*Mimosa intsia*	Root	Ayurveda	[29]
*Phoenix loureiroi*	Tender stem	Ayurveda	[29]
*Rhaphidophora pertusa*	Aerial roots and leaves	Ayurveda	[29]
*Symplocos loba*	Bark	Ayurveda	[30]
*Oryza sativa* Linn	Fruits and roots	Ayurveda	[31]
*Eurycoma longifolia*	Root	Malay	[32]
*Labisia pumila*	Leaves and roots	Malay	[33]
*Piper sarmentosum*	Leaves	Malay	[34]
